# Guidelines for inclusive and equitable energy and transport modeling

**DOI:** 10.1016/j.isci.2025.113218

**Published:** 2025-07-28

**Authors:** Marissa Bergman, Julia Tomei, Stephanie Hirmer, Beatrice Stockport, Fatima Afifah, James Dixon, Leonhard Hofbauer, Alycia Leonard, Pietro Lubello, Elena Pierard Manzano, Brunilde Verrier, Margaux Daly, Neve Fields, Francesco Gardumi, Steve Pye, Mourice Kausya, Kirsty Mackinlay, Kevin Nayema, Elsie Onsongo, Divya Subash Kumar

**Affiliations:** 1 Institute for Sustainable Resources, University College London, London WC1H 0NN, UK; 2 Department of Engineering Science, University of Oxford, Oxford OX1 3PJ, UK; 3 Department of Civil & Environmental Engineering, University of Strathclyde, Glasgow G1 1XJ, UK; 4 UCL Energy Institute, University College London, London WC1H 0NN, UK; 5 Transport Studies Unit, School of Geography and the Environment, University of Oxford, Oxford OX1 3QY, UK; 6 STEER Centre, Loughborough University, Loughborough LE11 3TU, UK; 7 Department of Energy Technology, KTH Royal Institute of Technology, 100 44 Stockholm, Sweden; 8 Nuvoni Centre for Innovation Research, Nairobi, Kenya; 9 Centre for Global Equality, Cambridge CB1 1EL, UK; 10 Department of Chemical Engineering and Biotechnology, University of Cambridge, Cambridge CB3 0AS, UK; 11 Centre for Climate Change and Disaster Management, Anna University, Chennai 600025, India

**Keywords:** Energy resources, Energy policy

## Abstract

Energy and transport models are powerful tools for shaping policy, development pathways, and financial decisions. However, these models often fail to account for gender equality and social inclusion (GESI), perpetuating systemic inequities and excluding the needs of marginalized communities. This perspective presents guidelines, developed through a collaborative process informed by a scoping literature review and expert consultation with modelers and social scientists, for integrating GESI into large-scale energy and transport systems modeling processes, particularly in low- and lower middle-income countries. By addressing key challenges—such as data disaggregation, the limits of current model architectures, and the complexities of quantifying social factors—we outline steps to incorporate GESI considerations throughout every stage of the modeling life cycle. While developed with energy and transport systems in mind, the principles of these guidelines are broadly applicable to other infrastructure modeling domains. Ultimately, this work demonstrates how inclusive modeling practices can produce more equitable, context-sensitive results, and foster sustainable development outcomes.

## Graphical abstract

**Figure:**
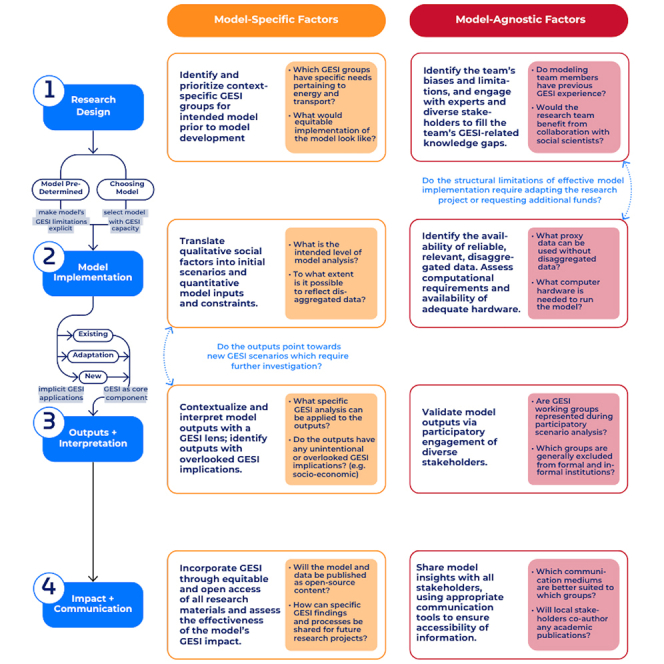

## Introduction

Large-scale systems infrastructure development creates both benefits and burdens—and the ways in which these are distributed can lead to inequality and injustice.^1^^,^^2^ While universally equitable distribution of benefits is unrealistic, it is disproportionately marginalized and vulnerable groups who benefit the least and bear the greatest burden.^3^^,^^4^ Energy and transport systems, as vital infrastructures and societal cornerstones, are particularly prone to reinforcing these inequities if poorly planned, as they shape access to economic opportunities and essential services.^5^^,^^6^

Gender equality and social inclusion (GESI) is a concept aimed at eliminating barriers and enhancing participation for marginalized and vulnerable populations.^7^^,^^8^^,^^9^ As outlined in Table 1, unlike broader frameworks of energy and transport justice, which emphasize equitable access across systems, GESI specifically prioritizes the unique needs of marginalized groups.^22^ While GESI and justice frameworks intersect and share common principles, focusing only on justice without explicit consideration of GESI risks overlooking the complex, intersecting vulnerabilities of social exclusion.^23^ Ignoring GESI in this context perpetuates inequity by implying that all individuals experience energy and transport systems uniformly, thereby allowing systemic disparities to persist.^24^^,^^25^

**Table 1:** Key GESI groups in LMICs and a sample of their intersections with energy and transport modeling

Key GESI group	Energy: Unique needs and vulnerabilities	Transport: Unique needs and vulnerabilities	Relevance to energy and transport modeling
Children and youth	Lack of reliable electricity for studying after dark; energy needs for educational purposes (e.g., digital tools)	Traveling to school and play areas can be unsafe (e.g., lack of designated crossings and paths)	Modeling exercises can overlook how energy access—or lack thereof—affects children’s educational success in energy-insecure households. Model scenarios can explore stop-gap measures, highlighting the broader societal benefits of improved education outcomes.
LGBTQIA+	Disproportionately energy insecure due to housing instability	Risk of harassment or violence while utilizing transport; exclusion from transport	Transport models typically assume universal safety; integrating security metrics as model inputs (e.g., improved lighting on vehicles), could enhance security.
Migrants and refugees	Barriers to formal energy access due to legal status; refugee camps’ energy access led by humanitarian aid	Restricted access to formal transport due to legal status; refugee camps isolated from urban centers	Energy models rarely account for displacement, assuming stable populations, or humanitarian-led energy provisions. Integrating decentralized energy scenarios (e.g., solar mini-grids in refugee camps) could highlight potential policy interventions.
Older adults	Physical limitations affecting energy use (e.g., heating and cooling needs)	Mobility constraints and inaccessible public transport; reliance on family assistance	Accessibility is not often a factor in transport models. Revising model inputs to reflect longer pedestrian crossings will accommodate slower walking paces and wheelchair users.
People with disabilities	Reliable electricity for assistive devices (e.g., screen readers, hearing aids); resultant higher energy costs	Inaccessible transport systems (e.g., lack of ramps, auditory cues, tactile pavers at crossings)	Models rarely include the energy needs of medical and assistive technologies, or care facilities. Inputs reflecting this higher energy load demand will better enable equitable results.
People living in informal settlements	Generally off-grid with informal connections, which are unreliable, costly, and hazardous	Limited access to formal transport; reliance on unsafe or informal transport for essential services	Energy models often neglect informal grids, focusing on formal infrastructure. Unregulated, unmetered energy markets are frequently controlled by cartels, leading to exploitation and abuse of vulnerable populations. Models can reflect informal energy systems to generate results highlighting expansion of safe and reliable energy.
People living in poverty	Reliance on inefficient and unsafe sources (e.g., kerosene); energy poverty perpetuates cycles of poverty	Unable to afford public transport; more likely to depend on non-motorized transport, “NMT” (e.g., walking and cycling)	Transport models often focus on motorized transport infrastructure, favoring users who can afford public or private transport. Models could incorporate disaggregated NMT-use data to simulate scenarios centering NMT infrastructure development.
Rural communities	Lack of reliable energy infrastructure; dependence on traditional fuels (e.g., biomass) with health hazards	Isolation and limited public transport; dependence on informal transport; longer travel times and higher costs	Cost-optimization energy models often prioritize urban areas due to higher population density, compared to less inhabited rural areas. Incorporating spatially granular data in models can highlight infrastructure gaps for model recommendations expanding rural electrification.
Women and girls	Gendered disparities in energy poverty; domestic and caregiving responsibilities; resultant physical and mental health risks and educational barriers	Gendered time poverty driven by caregiving travel patterns; risk of harassment or violence while utilizing public transport	Transport models prioritizing rush-hour commuters neglect the mobility patterns of women working in the social care sector, who travel during off-peak hours or make multiple stops during excursions. This oversight yields gendered time poverty and higher costs for multi-modal trips. Poor transport accessibility has the potential for cascading economic disruptions in sectors reliant upon care workers.

An inexhaustive list of factors by which groups can be marginalized includes age, caste, class, disability, ethnicity, gender, indigeneity, informal settlement status, migration status, race, refugee status, religion, rurality, sexuality, and socio-economic status (including living in poverty). We acknowledge that marginalization can occur across a broad range of factors, often in intersecting and context-specific ways. Sourced from the literature and authors’ expertise.^3^^,^^4^^,^^5^^,^^6^^,^^7^^,^^10^^,^^11^^,^^12^^,^^13^^,^^14^^,^^15^^,^^16^^,^^17^^,^^18^^,^^19^^,^^20^^,^^21^

Modeling commonly underpins infrastructure planning, generating insights that influence policy, shape development pathways, and direct financial capital.^10^^,^^26^ As the energy and transport sectors are shaped by socio-political conditions, models representing these systems should incorporate such factors to reflect how they function in reality.^27^ Models that fail to consider this nuance can generate results that are inadequate, incomplete, or inaccurate.^28^ In particular, ignoring GESI in modeling exercises risks producing uniquely misleading outputs that entrench social inequalities and, in the worst cases, exacerbate vulnerabilities—creating broader systemic consequences through feedback loops.^29^

Conversely, embedding GESI into the early stages of energy and transport planning, by reflecting these considerations in modeling processes, will offer policymakers valuable insights into social inclusion when making decisions about infrastructure implementation.^30^^,^^31^ Applied conscientiously, this can foster net zero-aligned energy and transport development, supporting efforts to limit global warming to 1.5^O^C, while advancing a just transition that ensures marginalized communities are not left behind in the pursuit of sustainable development.^11^^,^^25^

Yet, most energy and transport models overlook social inclusion or treat GESI as an afterthought.^32^ This notable lack of representation perpetuates structural inequalities and limits the ability of models to address salient social challenges.^33^ To date, there has been no systematic effort to integrate GESI into all aspects of the modeling process—from design to data collection, scenario development, and resultant policy recommendations.

Here, we offer a perspective that addresses a critical gap in research and practice by advocating for the integration of GESI into large-scale energy and transport modeling to support sustainable development in low- and lower middle-income countries (LMICs).^34^ We present comprehensive guidelines to embed GESI throughout such modeling processes, giving consideration to equity concerns at every stage of the modeling life cycle, marking an essential step toward transformative change in just infrastructure planning.

## Context: Current state of GESI in energy and transport modeling processes

Building upon the outlined potential of GESI to enhance modeling practices, we undertook a scoping literature review to assess the current state of GESI integration in energy and transport models.^11^ Our review was not limited to any single modeling methodology, as energy and transport researchers employ a range of modeling approaches. Historically, these have centered on techno-economic models, which optimize or simulate systems primarily based on cost, technology choices, and infrastructure deployment.^35^^,^^36^ In energy, these include integrated assessment models (IAMs) and geospatial electrification tools, while in transport they encompass network optimization and travel demand models, with cross-sector tools like cost-benefit analyses frequently applied across both domains.^30^^,^^37^ More recently, socio-technical modeling approaches have emerged, aiming to capture social, political, and behavioral dynamics alongside technological and economic considerations.^38^^,^^39^ Such methods include system dynamics (SD) modeling, which uses feedback loops to represent nonlinear and complex interdependencies, and agent-based models (ABMs), which simulate decisions and interactions of individual actors or communities.^17^^,^^27^^,^^40^

Given that much of the energy and transport infrastructure development work is driven by international development organizations, our review encompassed both academic literature and gray literature from these entities, such as the United Nations Office for Project Services (UNOPs) and United States Agency for International Development (USAID), to capture a comprehensive view of GESI integration efforts.^41^^,^^42^^,^^43^ Ultimately, our review of over 80 pieces of literature revealed a striking knowledge gap regarding the incorporation of social inclusion across all large-scale systems infrastructure modeling processes.^29^^,^^44^ Due to this scarcity, we expanded our scope beyond energy and transport to include other infrastructure domains, seeking insights from related sectors.^45^ The most extensive guidance identified in our review comes from a non-academic source: the Australian Aid-funded toolkit on GESI in water modeling for Nepal’s Water and Energy Commission—work that, while valuable and informative, falls outside the scope of energy and transport systems.^46^

In parallel to the literature review, we engaged in consultation with 26 energy and transport modelers and social scientists specializing in GESI to further contextualize the current practices and limitations in reflecting social inclusion in modeling tools. The findings from these discussions enriched the landscape assessment presented here and provided critical input that informed the initial creation of our guidelines. Of these, 10 engagements were exploratory conversations to understand broad perspectives in the field, while 16 experts subsequently contributed to collaborative workshops and iterative discussions to co-develop the guidelines, and ultimately joined this manuscript as authors.

## GESI knowledge gaps

We found that, while academic literature broadly acknowledges the importance of social inclusion in energy and transport development, few studies address how social inclusion can be represented within modeling exercises.^47^^,^^48^^,^^49^ Notable exceptions include reviews by Lonergan et al. and Vågerö and Zeyringer, which confirm the justice gaps in energy models; proposing solutions is, however, outside the scope of work.^11^^,^^31^ The perspective by Dioha et al. emphasizes the critical need to integrate socio-political factors into models and proposes developing new metrics, linking different model types (also known as “soft-linking”), and fostering interdisciplinary collaboration—concepts that align with insights from our consultations and workshops and are reflected in our guidelines.^33^ While these studies collectively underscore the importance of GESI considerations in modeling, none provide a systematic framework for integration throughout the modeling life cycle.

Beyond these exceptions, several studies engage with related concepts but are tangential to the representation of GESI in modeling. Goforth et al.’s work provides explicit guidance on research requirements to incorporate energy justice into power systems modeling, criticizing the lack of useful quantitative metrics.^50^ However, this work addresses issues of justice more broadly, rather than focusing on the specific concerns of vulnerable populations. Similarly, the research of Cherp et al. reflects individuals’ decision-making in relation to equitable energy access, without addressing the distributional impacts of infrastructure systems on marginalized groups.^35^ Other work, such as Trutnevyte et al., frames social inclusion as an external factor to consider during the interpretation of model results, rather than as an integral component of the model architecture.^37^ This delineation of consequence, exogenous versus endogenous, can create inherent biases against GESI in model outputs.^10^

The gap in GESI integration exists partly because qualitative social factors fall outside the remit and expertise of quantitative modelers, and it is unfair to expect them to address these complexities alone.^39^^,^^40^ Collaboration with social scientists offers a compelling remedy to the dearth of GESI considerations in energy and transport modeling processes.^38^^,^^51^ Ultimately, the lack of interdisciplinary engagement underscores the necessity and urgency of our guidelines, which were co-created through a collaboration between multidisciplinary academics and practitioners to bridge this gap. The following section explores why such work has been underexplored and the challenges that hinder its inclusion in modeling practices.

## Challenge framing: Limitations of incorporating GESI in modeling processes

In developing our guidelines, we identified three consistent barriers to effectively incorporating GESI in modeling processes. These barriers emerged from the literature review and engagement with energy and transport modelers, offering both theoretical and practical insights into the challenges that persist. The key barriers identified, though not exhaustive, are as follows.

### Model capabilities and objectives

Not all models are well-suited to reflect GESI, due to their internal logic and resolution of analysis. For example, linear optimization modeling is not built to reflect social factors, which are nonlinear, and spatially aggregated models do not reflect localized, granular inputs or outputs—an inherent conflict with the context-specific nature of GESI.^17^^,^^31^^,^^50^ The level of disaggregation also applies to demographics; when a population is considered in aggregate in modeling, distributional impacts on vulnerable groups can be obscured.^52^ Additionally, supply-side focused models rely on exogenous demand assumptions, limiting their ability to parameterize the social impacts of upstream decisions.^32^

While these factors make it difficult to incorporate GESI as a core component of energy and transport models, it is possible to translate qualitative factors into quantitative metrics—within reasonable limits.^40^^,^^53^ Forcing nuanced social dynamics into ill-equipped models can undermine the intended GESI insights and threaten the veracity of model results by stretching its purpose.^33^^,^^44^

### Data availability, reliability, and ethics

A model’s ability to reflect GESI can also be limited by a lack of relevant data. This is a global issue, but can be especially challenging within LMIC contexts, in which disaggregated data are often unavailable and data around marginalized communities unreliable.^10^ For instance, people with disabilities and LGBTQIA+ populations are at risk of violence or legal punishment in numerous LMICs, and are therefore prone to vast underreporting and privacy concerns in data collection.^52^ These privacy risks are not merely technical barriers, but raise fundamental data justice concerns: even high-level demographic data, if mishandled, can expose vulnerable groups to disproportionate harm, placing a particular ethical responsibility on researchers to safeguard such information.^54^^,^^55^

However, issues of data are more complex than readiness and reliability; indeed, social inequalities in energy and transport often remain hidden even within disaggregated datasets.^56^ For example, gender-disaggregated data fails to capture the intrahousehold dynamics of energy usage, in which men frequently benefit more from electrification than women.^3^^,^^57^

### Model complexity and computational resources

Even when a model has ready access to quality data and can effectively reflect social factors in its functionality, computational processes are not without limitations.^34^ For example, the more variables included in a model, as required to accurately reflect GESI, the more complicated and unwieldy it becomes. This is counterproductive if a research objective is to produce models as user-friendly tools.^15^ Similarly, higher complexity requires longer runtimes and increased computing power to handle processing, both of which can make the model exclusionary in LMICs, where technocrats and planners often face resource constraints.^26^^,^^46^ In such contexts, factors like unreliable electricity, inadequate internet connectivity, and limited digital infrastructure pose computational barriers and raise ethical considerations around how scarce resources are allocated between advanced modeling efforts and development needs.^58^

While these three challenges present legitimate constraints to GESI integration in energy and transport modeling, we argue that they should not deter efforts to pursue more inclusive practices. Our guidelines acknowledge these limitations and emphasize the importance of identifying and transparently communicating them, enabling resources to be allocated for innovative solutions. We believe that recognizing the boundaries of GESI integration within a project is not a constraint but an opportunity for creative problem-solving in the pursuit of equitable infrastructure development. Indeed, researchers have successfully used unconventional approaches—such as analyzing mobile phone data to capture transportation patterns—to approximate social factors where traditional methods fall short.^59^ We encourage embracing such ingenuity to support the integration of GESI considerations in difficult modeling contexts.

## Solution: Guidelines for incorporating GESI in energy and transport systems modeling processes

Here, we propose guidelines for incorporating GESI considerations in energy and transport systems modeling processes. Importantly, GESI integration exists on a spectrum—from the minimum do-no-harm principle, to limiting social exclusion, to actively designing models that provide insights for improving the livelihoods of marginalized populations. We recognize that implementing these guidelines are a significant undertaking for modeling teams and do not expect every recommendation to be feasible in every context. Instead, we encourage practitioners to engage critically, prioritize actions, and adopt what is practical for their specific circumstances, while remaining open to pushing boundaries and challenging the status quo. Crucially, achieving meaningful GESI outcomes cannot rest on modelers alone; close collaboration with social scientists and stakeholders is requisite to bridge disciplinary gaps.

Following the initial consultations and draft, these guidelines were co-developed among the authorship, iteratively refined through workshops and discussions with both modelers and social scientists with GESI expertise. This group reflects diverse and complementary modeling approaches working on different scales, including practitioners working with techno-economic optimization models (e.g., OSeMOSYS), energy demand analyses (e.g., MAED), transport system simulations (e.g., TEAM), integrated resource assessments (e.g., CLEWs), geospatial modeling techniques (e.g., OnSSET and GeoH2), and emerging SD approaches for socio-technical transitions.^27^^,^^60^^,^^61^^,^^62^^,^^63^^,^^64^^,^^65^^,^^66^^,^^67^ This collaborative, robust process and cross-checking ensured that the guidelines remain flexible and relevant across a wide range of modeling methodologies.

**Figure 1:**
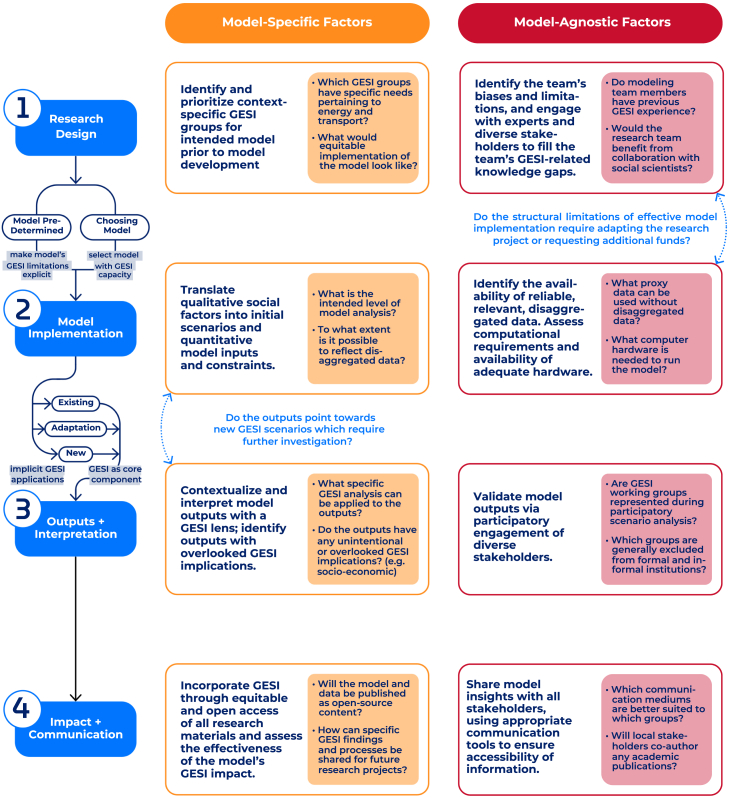
Flowchart of guidelines for incorporating GESI in energy and transport systems modeling processes

Visualized as a flowchart in Figure 1, illustrated through a case study in Box 1, and outlined in full in Table S1, our guidelines present a series of questions designed to direct modeling teams through the incorporation of GESI. The questions serve a dual purpose: prompting critical reflection and discussion around research practices, while also offering practical recommendations on ways to execute GESI-centered ideation, where possible. The guidelines are intentionally broad, rather than prescriptive, as the reflection of GESI in modeling depends on the model itself, research objectives, geographic context, and marginalized groups impacted.

Box 1 **Illustrative example of applying GESI guidelines to GeoH2**

To illustrate the practical application of our guidelines, we present a retrospective analysis of the GeoH2 model, a geospatial cost-optimization tool designed to identify locations for green hydrogen production, storage, and transportation. It has been used to assess the feasibility of large-scale hydrogen deployment in various contexts (e.g., Kenya and Namibia).^68^^,^^69^^,^^70^ While the model was not originally developed with GESI considerations, this example demonstrates how the systematic adoption of our guidelines could have enhanced the integration of GESI across all stages of the modeling process.

At the research design phase (Stage 1), economic efficiency was prioritized within the optimization framework to produce a “minimum viable” model, while considerations of equitable outcomes were left to be implemented later. Had GESI been embedded from the outset, hydrogen site selection criteria could have incorporated metrics such as workforce access, land tenure risks, or equitable infrastructure distribution to enable a broader range of communities to benefit from hydrogen expansion.

During model implementation and interpretation (Stages 2 and 3), the focus on minimizing costs resulted in outputs that heavily prioritized areas with high technical renewable potential, irrespective of their social makeup or impacts of development. For instance, in Kenya, regions near Lake Turkana show up as highly promising, despite ongoing tension with local communities over renewable development. This has the potential to reinforce existing inequalities, as social factors do not play into the core optimization. While layering demographic and socio-economic data during model interpretation exposes these disparities and informs more inclusive recommendations that explicitly target underserved populations, these could also be baked into the optimization itself through improved model implementation. Moreover, the GeoH2 model inputs could have been adjusted to prioritize access for rural communities, for instance by introducing a social cost or benefit based on co-electrification. Crucially, our guidelines emphasize how insights from interpretation can feedback into model implementation, prompting refinements to the input assumptions, constraints, or optimization goals to iteratively improve GESI outcomes.

While GeoH2’s findings were communicated primarily through technical and economic reporting (Stage 4), a GESI-oriented dissemination strategy could have highlighted the distributional impacts of hydrogen infrastructure, guiding more inclusive policymaking.

Overall, this retrospective application illustrates that even models not originally designed with GESI in mind can be strengthened by our guidelines. By systematically identifying practical entry points and “low-hanging fruit,” researchers can enhance social inclusion without requiring model redesign.

While we recommend applying the guidelines from the pre-development stage for comprehensive inclusion of GESI, its modular approach enables adoption at any stage of the modeling process. Within each phase, we distinguish between two sets of factors:

• ***Model-specific*** factors encapsulate the ways in which GESI can be embedded directly into the modeling process: design, implementation, interpretation, and dissemination of results.
• ***Model-agnostic*** factors consider the systems surrounding the model, such as research teams, academic institutions, funding mechanisms, and stakeholder engagement practices.

Neither component should be prioritized over the other; instead, both should be considered in parallel, as they are inherently intertwined and influence one another.

Notably, one aim of incorporating GESI into modeling processes is to empower local stakeholders, whose preferences and limitations need to be considered, rather than defaulting to only prioritizing research-driven agendas.^71^ There may be varying levels of appetite for redressing inequalities among stakeholders and particularly so regarding marginalized groups.^21^ When utilizing these guidelines, it is imperative to avoid implementing well-intended but context-inappropriate GESI solutions.^72^ Ultimately, while GESI integration in modeling can drive more inclusive outcomes, it cannot, on its own, dismantle the socio-political structures that perpetuate social exclusion.^39^

### Stage 1: Research design

Centering GESI as a fundamental element of research design lays the foundation for successful social inclusion throughout the modeling process, as decisions made at this stage shape the potential for GESI integration in subsequent phases.^46^ This necessitates acknowledging the team’s GESI limitations and engaging with experts and diverse stakeholders to augment such gaps, building an inclusive team, and amplifying marginalized voices.^51^^,^^73^ However, giving proper credence to GESI requires additional resources, such as the cost of engaging experts or the increased time necessary for participatory processes and trans-disciplinary collaboration, hence the utility of charting these needs within the research funding structure.^2^

The extent to which GESI can be considered is related to the model’s architecture. There is more flexibility in choosing a model during the design of a research or infrastructure planning initiative: the model’s potential to reflect social inclusion can influence the selection or prompt the decision to create a new model. Conversely, if an existing model was pre-determined for utilization, this requires identifying its GESI limitations and developing workarounds, such as soft-linking multiple models with various specialties and functions (e.g., pairing a cost-optimization model with an SD model that can simulate group behaviors).^74^ Care should be given when models developed for high-income country (HIC) applications are utilized in LMICs, which have distinct energy and transport needs that may require adaptation of the model to be represented accurately.^75^

This is the ideal stage during which to identify context-specific GESI groups, so their needs and vulnerabilities can be studied prior to model implementation. No one GESI classification represents a homogenous group, and people can be concurrently marginalized by multiple factors (e.g., children living in poverty or refugees with disabilities), thereby facing even further discrimination.^56^^,^^76^^,^^77^ It is critical to understand how social exclusion is compounded by imbuing the model with an intersectional lens.^12^^,^^78^^,^^79^ Yet, logistical practicalities prevent engaging with or reflecting all marginalized groups in one model; we suggest researchers prioritize the most affected GESI groups based on the project goals and context, meaningfully engaging with critical issues rather than superficially touching many.^20^ This enables limited resources to be allocated where they can achieve the greatest impact. Once the most impacted groups have been identified, their prioritization can be made explicit; transparency about the limitations and trade-offs of incorporating GESI is essential to maintain research credibility.^80^

Instituting GESI considerations during the research design stage not only supports inclusivity from the outset but also establishes a clear precedent for the subsequent stages of model implementation, interpretation, and communication. Regardless of which stage the guidelines are first applied, identifying the appropriate GESI groups is indispensable; effectiveness hinges on this step, as the core purpose is to accommodate and address the needs of these groups.^46^

### Stage 2: Model implementation

Model implementation is the stage in which qualitative social factors are translated into quantitative terms—whether as inputs, constraints, or through the inclusive development of initial scenarios used to run the model.^38^^,^^50^ There is no universal method for quantifying GESI, nor would one be appropriate, as this translation necessarily depends on the context-specific insights, stakeholder engagement, and interdisciplinary collaboration established during Stage 1. Moreover, there are inherent risks in reducing social factors to quantitative metrics, as it may oversimplify complex dynamics, which must be balanced with the multidimensional model objectives, such as economic and environmental considerations.^81^

Examples from existing modeling work illustrate how such context-specific quantification can be approached. For instance, Trotter et al.’s energy planning in Uganda integrated urban-rural equity by imposing constraints in a multi-objective optimization model, to ensure the disparity in electrification rates between urban and rural areas did not exceed a specified limit.^82^ Menghwani et al. applied a geospatial least-cost electrification model, OnSSET, in a case study for Tanzania, focusing on people living in poverty.^30^ Using existing statistics on poverty rates to identify this GESI population within each geographic cell, electricity prices were adjusted so that overall revenues were redistributed; other users were charged higher prices to subsidize affordable rates for communities living in poverty.

The extent to which GESI can be reflected at this stage depends on both the model type and the research team’s control over its architecture, including aspects such as the spatial scale of analysis (e.g., national vs. sub-national).^35^^,^^50^ New models developed with GESI as a ***core component*** of their purpose, in which a key structural function of the model is representing social inclusion, can utilize such inputs directly, such as focusing expansion of public transport to historically segregated neighborhoods.^48^ Existing models with limited capacity for social inclusion might reflect GESI ***implicitly***, in which inputs and outputs include GESI dimensions as indirect by-products. For example, a model whose primary purpose is calculating the amount of electricity necessary to power expansive street lighting might not focus explicitly on social dynamics, but this output can reduce crimes and violence against women.^83^

Even when it seems impossible or irrelevant to consider social inclusion, modelers should question the underlying GESI biases in their model assumptions and attempt to rectify any such imbalances. For instance, energy models that focus on historical electricity consumption when determining future demand often assume higher levels in urbanization, due to the pre-existing electrical access, creating a disproportionate favor for urban centers regarding energy infrastructure, thereby marginalizing rural communities.^84^

As highlighted in the challenge framing section, the capacity to reflect GESI in model implementation is further influenced by agnostic factors, such as data readiness and computational capacity. In practice, rather than collecting new primary data, most modeling processes rely on existing national datasets, typically compiled by governments or international institutions, which are aggregated and lack detailed delineation of GESI groups. One practical strategy to address this challenge is the creative use of proxy data, whereby alternative indicators substitute for missing information. For example, researchers could use night-time satellite imagery to approximate electricity access in rural settlements.^85^

Model complexity poses unique challenges for those intended to be run in LMICs, where resource constraints can limit the feasibility of executing computationally demanding processes.^58^ Given that energy and transport infrastructure models are already intricate, incorporating extensive GESI dimensions can risk rendering them impractical for local use; instead, practitioners should prioritize a few high-impact GESI variables, such as urban-rural residence or poverty levels.^86^ One strategy to balance complexity with inclusivity is to adopt modular modeling approaches that allow additional GESI dimensions to be integrated incrementally without overburdening computational resources; the open-source OSeMOSYS tool, for example, offers flexible architecture while remaining accessible for use in LMIC contexts.^87^^,^^88^^,^^89^

The combination of model-specific and model-agnostic structural barriers can create a cycle, whereby limited resources constrain GESI integration in modeling projects, and this absence of GESI considerations perpetuates exclusion in future models and knowledge creation. Overcoming these barriers may require adapting the research scope or securing additional funding to prevent GESI considerations from being sidelined during implementation.^15^^,^^44^

### Stage 3: Outputs and interpretation

The third mode by which GESI can be reflected in modeling is ***interpretation***, which applies a social, political, and economic lens to the model outputs, especially when combined with participatory processes that engage diverse stakeholders.^37^ This approach is the most common way in which existing models reflect social inclusion, as it does not require material changes to the model architecture or quantitative constraints.^31^ However, while this stage is critical, it is not a substitute for embedding GESI throughout the entire modeling process—early integration enables inclusion to be built into the foundation rather than retrofitted at the end.

Beyond embedding GESI considerations into the model implementation scenarios, layering targeted GESI scenarios on top of model outputs can add depth by highlighting nuanced, context-specific insights, particularly for marginalized groups.^51^^,^^90^ For example, a public transport expansion model could be analyzed through the lens of proximity and service frequency to health centers, which support the empowerment of vulnerable groups, such as older adults, people with disabilities, and women and girls.^21^

Importantly, model outputs are not simply an endpoint but can inform new modeling iterations in a feedback loop to pursue under-investigated GESI dimensions. Continuing the example of transport access to health centers, identifying gaps in service coverage might prompt practitioners to adjust and re-run the model, introducing constraints to prioritize routes in underserved areas or reduce maximum travel times for affected groups. Such iterative refinement promotes models that evolve alongside stakeholder input and priorities.

Validation workshops can be used to capture stakeholder insights; it is valuable to engage participants representing a wide range of perspectives, as disregarding GESI in the participant pool can lead to outputs that fail to reflect true social inclusion.^2^^,^^18^ Tokenization of marginalized individuals can be avoided by engaging with working groups, whom are experts on these specific needs and vulnerabilities.^15^ Crucially, involving diverse stakeholders must go beyond a performative exercise; meaningful engagement requires actively listening to participants and ensuring both recognition and procedural justice.^34^^,^^91^

Further, stakeholder engagement should permeate the entire modeling process. While this perspective highlights its role in interpretation, participatory processes are equally critical in earlier stages.^92^ As outlined in our guidelines, involving stakeholders from the outset allows them to shape research methodologies and co-create scenario storylines for model implementation, further enriching the inclusivity of the outputs interpreted in this stage.

### Stage 4: Communication and impact

The final stage of any modeling project is disseminating insights to stakeholders. This audience can range from policymakers and funding bodies who utilize models to inform legislative and financial decision-making, to local communities impacted by such decisions, and the wider academic community referencing the research for future knowledge creation.^10^ Within the LMIC research remit, in which these models serve as tools for development and aid programs, there is a risk of perpetuating colonial dynamics and exerting undue influence.^73^^,^^75^^,^^93^ GESI considerations must therefore extend to how findings are shared, prioritizing transparency and accessibility; even the most inclusive models can fail to promote equity if the findings are dissonantly disseminated.

Communication strategies can explicitly address potential biases in how model outputs are presented, as each stakeholder group processes and values information differently and no one group’s preference should be favored.^93^ Research findings should be conveyed through appropriate channels and languages to maximize accessibility, clearly outlining how GESI factors were considered during the modeling process and clarifying trade-off decisions to foster trust.^94^^,^^95^

Notably, communication is not confined to final deliverables—it must be continuous and reciprocal throughout the entire research process.^34^ The most inclusive impact is achieved through sustained stakeholder engagement, even beyond the conclusion of the project.^22^ This might involve facilitating knowledge transfer workshops or co-developing action plans with impacted communities.^51^ Such participatory approaches not only validate the research but also empower stakeholders to utilize findings effectively.

In academia, inclusivity can be supported through open-access repositories of all modeling materials, including data and assumptions to enhance transparency and interoperability.^74^^,^^92^ However, this must not compromise the safety of GESI communities; anonymization and ethical safeguards may be necessary to protect vulnerable groups.^96^ Furthermore, open-access alone does not guarantee accessibility; poorly documented repositories with insufficient metadata remain inaccessible to the wider research community, while the high costs of publishing open-access can exclude researchers from low-resource settings.^26^

Finally, modeling processes would ideally culminate in a critical reflection on how the outcomes can inform and propel future research that explicitly advances GESI, helping new knowledge build progressively toward more equitable and inclusive systems. Such reflection should also encompass researchers’ own learning, recognizing how engaging with GESI perspectives can reshape their assumptions, practices, and priorities for future work.

## Outlook

Large-scale systems infrastructure modeling holds considerable influence over policy and investment decisions, carrying profound implications for social and environmental justice. Incorporating GESI into energy and transport modeling is not just a matter of equity, but a vital component of achieving sustainable development goals, ensuring that infrastructure transitions are just, inclusive, and resilient in the face of global challenges. This perspective demonstrates how GESI can be systematically incorporated into energy and transport modeling, providing both a theoretical foundation and practical roadmap. As one of the first structured efforts of its kind, our guidelines challenge researchers, policymakers, and practitioners to rethink the role of modeling in advancing equitable infrastructure. Created through interdisciplinary collaboration between modelers and social scientists, the guidelines exemplify the integrated approach necessary to tackle the complex, interconnected challenges of sustainable development.

While our guidelines outline practical steps for embedding GESI at various stages of the modeling process, implementation requires a deliberate commitment to inclusivity and adaptability. The integration of GESI in modeling is not without challenges; however, by explicitly addressing these and continuously refining the research process, modelers can enhance the relevance and utility of their work. A nuanced and balanced model that considers GESI factors will generate outputs that not only account for social equity, but also better reflect real-world conditions, ultimately enhancing their robustness.

Incorporating GESI in modeling processes requires considered trade-offs: prioritizing the most impacted marginalized communities, balancing the complexity of social factors with data constraints, and weighing subjective inputs against more readily quantifiable metrics. There is no perfect execution of our guidelines; no single modeling exercise can address every dimension of GESI. Rather, the priority lies in maintaining transparency about these trade-offs. Energy and transport modeling carries an inherent responsibility; these tools influence financial and governance decisions that affect lives and communities. Modelers cannot plead neutrality or detachment in the face of such consequences.

Building on this commitment to equity and collaboration, there is considerable scope for future research stemming from our guidelines. They are not a rigid methodology, but, rather, an aspirational, structured framework intended to influence practice and inspire further inquiry; we view them as a living document to be revised and strengthened through continued learning and application. This work raises questions about the extent to which GESI can and should be embedded into models endogenously versus exogenously, with each approach offering benefits and challenges. We recommend testing and adjusting the guidelines through case studies, both retrospectively to identify entry points for inclusion in completed modeling exercises and in partnership with ongoing projects to integrate GESI considerations throughout the research life cycle. While the guidelines were developed for energy and transport modeling, the broader principles are applicable outside these spheres and can be adapted to other domains of infrastructure modeling, encouraging cross-sectoral application and innovation.

We acknowledge that the majority of this perspective’s authors are affiliated with academic institutions in HICs. While our guidelines are designed to support infrastructure development in LMICs, we recognize the complex power dynamics inherent in HIC-led research. Our aim is to contribute to more equitable infrastructure modeling by centering GESI considerations, and we encourage further collaborations that elevate voices from LMICs in shaping these research agendas. Supporting climate-resilient development, particularly in regions where the impacts of climate change are disproportionately felt, requires continuous reflection, inclusivity, and genuine partnerships across geographies and disciplines.

## Acknowledgments

This material has been produced under the 10.13039/100018066Climate Compatible Growth (CCG) program, which is funded by the UK’s Foreign Development and Commonwealth Office (10.13039/501100020171FCDO). However, any views expressed herein do not necessarily reflect the UK government’s official policies. We would like to thank Sarel Greyling for designing the flowchart of the guidelines and Jennifer Cronin, Mark Howells, Nick Hughes, Hannah Luscombe, Ariane Millot, James Price, Jairo Quiros-Tortos, Vignesh Sridharan, Naomi Tan, and Rudolf Yeganyan for their feedback and support.

## Author contributions

M.B.: conceptualization, writing – original draft, writing – review & editing, project management; J.T., S.H., and B.T.: conceptualization, writing – review & editing, project management; F.A., J.D., L.H., A.L., P.L., E.P.M., B.V., M.D., N.F., F.G., and S.P.: conceptualization and writing – review & editing; M.K., K.M., K.N., E.O., and D.S.K.: conceptualization.

## Declaration of interests

The authors declare no competing interests.
