# Supplementary material for: Guidelines for inclusive and equitable energy and transport modeling
Source: iScience. 2025 Jul 28;28(9):113218. doi: 10.1016/j.isci.2025.113218 (PMC12441686; doi:10.1016/j.isci.2025.113218)
Supplement: Document S1. Table S1 [file mmc1.pdf]

iScience, Volume *28*

Supplemental information

Guidelines for inclusive and equitable energy and transport modeling

Marissa Bergman, Julia Tomei, Stephanie Hirmer, Beatrice Stockport, Fatima Afifah, James Dixon, Leonhard Hofbauer, Alycia Leonard, Pietro Lubello, Elena Pierard Manzano, Brunilde Verrier, Margaux Daly, Neve Fields, Francesco Gardumi, Steve Pye, Mourice Kausya, Kirsty Mackinlay, Kevin Nayema, Elsie Onsongo, and Divya Subash Kumar

Table S1. Guidelines for incorporating GESI in energy and transport systems modeling processes

Stage	Factor	Question
**Research Design**	Model-Agnostic	Do modeling team members have adequate GESI expertise? If not, will external GESI experts be utilized?
		Are representatives from GESI working groups consulted during the research design process? Are local stakeholders involved in the co-creation of the research questions?
		Would the research team and objectives benefit from collaboration with social scientists?
		Is the research team itself inclusive, with members representative of GESI?
		What support and resources are necessary to successfully implement GESI in modeling? How can these needs be integrated into the research design and funding processes?
	Model-Specific	Is part of the research design process selecting or developing a model, or is the project locked into an existing, pre-determined model? If the former, does the ability for GESI representation influence the model selection? If the latter, how will you make the GESI limitations of the model explicit? Are there any workarounds for blind spots (e.g., soft-linking multiple models)?
		What are the research objectives and purpose of the model? To what extent is GESI relevant to these?
		**Identifying relevant GESI groups and prioritizing the most impacted:**
		Who are the research's main stakeholders? Which GESI groups are therein underrepresented?
		Do-No-Harm: What are the potential harmful consequences of the model objectives to GESI groups (e.g., what are their vulnerabilities?)
		Equity: What would equitable implementation of the model aims look like? (e.g., which groups are likely to be excluded from the model's benefits, and why?; which groups have unique needs pertaining to energy and transport?)
		Empowerment: In what ways can the model be utilized to actively improve the standing of GESI populations?
		How do the various components of GESI intersect? Will these needs and vulnerabilities change over time?
**Model Implementation**	Model-Agnostic	Is disaggregated GESI data available for this model and context? If not, what proxy data can be utilized? What other spatial data can give GESI insights into the modeling?
		In what ways can social exclusion be hidden in quantitative data (e.g., intrahousehold energy usage between genders)? What qualitative data is required to augment the quantitative, and better understand GESI in this context?
		How can comprehensive GESI data be gathered and protected for future modeling?
		What is needed in terms of computer hardware to run the model?
	Model-Specific	What is the intended level of model analysis? To what extent is it possible to reflect disaggregated data?
		Where is/was the model originally developed? If developed or originally utilized in a HIC context, how has/will the model be adapted for LMIC-specificity?
		Has GESI been factored into the initial scenarios utilized to run the model?
		How can the model remain user-friendly while representing GESI through additional levers?
		**Developing a New Model:** How can the model be developed in a way to center GESI as a **core component** of its function?
		**Adapting an Existing Model (adding new elements):** What elements of social inclusion were overlooked by the original model objectives? How can the model be adapted in a way to incorporate GESI into its new objective, either as a **core component** or **implicitly**?
		**Utilizing an Existing Model (only altering model inputs):** What elements of social inclusion are overlooked by the model objectives? How can GESI be reflected **implicitly** utilizing the existing parameters of the model?
Feedback Loop to Research Design		***Do the structural limitations of effective model implementation require adapting the research project or requesting additional funds?***
**Outputs + Interpretation**	Model-Agnostic	Is the group of stakeholders consulted during scenario development diverse? Are GESI working groups represented during participatory scenario analysis?
		How much power do GESI groups have in decision-making processes utilizing the model? Which groups are generally excluded from formal and informal institutions?
	Model-Specific	What specific GESI analysis can be applied to model outputs through further scenarios and narratives?
		Do the outputs have any unintentional or overlooked GESI implications? (e.g., socio-economic inclusion is considered GESI)
		What local expertise can be applied to the model outputs?

Feedback Loop to Model Implementation		***Do the outputs point towards new GESI scenarios which require further investigation?***
**Communication + Impact**	Model-Agnostic	How can the model insights be effectively communicated to different stakeholders? Which mediums and languages are better suited to which groups?
		Will local stakeholders co-author any academic publications or co-design other materials?
		Is there an internal or external evaluation mechanism to analyze the project's GESI accomplishments?
	Model-Specific	Will the model and data utilized be published as open-source content with ethical safeguards?
		Is there a way to share specific GESI findings and processes for future reference and research projects?
After Concluding		***How can future research create knowledge that explicitly advances GESI using this work?***
